# Benefits and detriments of interdisciplinarity on early career scientists’ performance. An author-level approach for U.S. physicists and psychologists

**DOI:** 10.1371/journal.pone.0269991

**Published:** 2022-06-30

**Authors:** Saïd Unger, Lukas Erhard, Oliver Wieczorek, Christian Koß, Jan Riebling, Raphael H. Heiberger

**Affiliations:** 1 Institute for Social Sciences, University of Stuttgart, Stuttgart, BW, Germany; 2 International Centre for Higher Education Research, University of Kassel, Kassel, HE, Germany; 3 Professorate for the Theory of Society and Comparative Macrosociology, Zeppelin University Friedrichshafen, Friedrichshafen, BW, Germany; 4 Human and Social Sciences Department, University of Wuppertal, Wuppertal, NRW, Germany; Iowa State University, UNITED STATES

## Abstract

Is the pursuit of interdisciplinary or innovative research beneficial or detrimental for the impact of early career researchers? We focus on young scholars as they represent an understudied population who have yet to secure a place within academia. Which effects promise higher scientific recognition (i.e., citations) is therefore crucial for the high-stakes decisions young researchers face. To capture these effects, we introduce measurements for interdisciplinarity and novelty that can be applied to a researcher’s career. In contrast to previous studies investigating research impact on the paper level, hence, our paper focuses on a career perspective (i.e., the level of authors). To consider different disciplinary cultures, we utilize a comprehensive dataset on U.S. physicists (*n* = 4003) and psychologists (*n* = 4097), who graduated between 2008 and 2012, and traced their publication records. Our results indicate that conducting interdisciplinary research as an early career researcher in physics is beneficial, while it is negatively associated with research impact in psychology. In both fields, physics and psychology, early career researchers focusing on novel combinations of existing knowledge are associated with higher future impact. Taking some risks by deviating to a certain degree from mainstream paradigms seems therefore like a rewarding strategy for young scholars.

## Introduction

Being highly productive and publishing many papers is a well-established factor for the success of academic careers [1–5]. Although recent research reveals that a consistent publication record moderates the “publish or perish” imperative [6], the pressure to publish is especially high for early career researchers, who yet have to leave a mark on the scientific discourse, and secure a position in a highly competitive job market [7–9]. We ask if it is beneficial for early career researchers to specialize in a research branch covered by a lot of other scholars or if they should pursue a novel line of research, which might yield high returns at later points in time. Should they pursue interdisciplinary research to maximize their potential audience and to boost their impact? To trace the impact of both strategies across multiple papers (i.e., authors’ publication records) is important, because it reveals (dis)advantages accumulated *by individuals*; and ultimately, scientists are the main driver of science’s dynamics [68].

To investigate research strategies like pursuing novel or interdisciplinary lines of research from a career perspective is complicated though. This is especially true in the early career phase, when researchers develop their personal research agenda [6, 10]. These difficulties may be the reason why previous studies focused mainly on articles to investigate how novelty and interdisciplinary research (IDR) affect research impact. Some researchers find positive effects of novelty and interdisciplinarity on scientific impact of articles measured in citation count [11–16], others stress negative effects of interdisciplinarity or novelty on research performance and research impact [17–19] or report ambivalent findings [20]. Still others focus on the degree of novelty in research pursued by early career researchers, but do not link these findings to scientific recognition in form of citations [21]. In contrast to previous studies concentrating on scientific *papers*, we look at the impact of research strategies from a career perspective to understand if and how they pay off for individual researchers in terms of increased citations and a larger impact. In so doing, we address the ambivalence in findings regarding the impact of interdisciplinarity and novelty, and expand the research beyond small-scale bibliometric datasets or surveys.

Because of their insecure position in the academic job market [5, 22], and their need to secure a place within the academic discourse, stakes are extremely high for early career researchers (ECR) and, hence, research strategies yield particularly important consequences. In the years following a PhD, ECRs who seek to remain in academia are faced with the task of making their mark in the highly competitive arena of academia. To achieve this, they must 1) develop a research identity suitable for a specific field of research [6, 23, 24], 2) find their own research agenda and become an independent researcher [25–27], and 3) contribute genuinely new insights to the academic community [6, 28]. In other words, if they conduct interdisciplinary and novel research, which might be highly risky research strategies in themselves, they have to do so in the most vulnerable phase of their academic career. We combine the early career perspective with the aforementioned research strategies to investigate the following questions: *Is it beneficial for the research impact of ECRs to 1) conduct interdisciplinary research, and/or 2) pursue novel lines of research?* Despite a large number of studies that addressed these questions before [21, 29–36], there are, to the best of our knowledge, no studies that tackle these questions a) with a large dataset of early career publication records and b) by comparing two disciplines at the same time.

To answer these research questions, we use a dataset of ECRs who completed their doctorates in physics or psychology at US universities between 2008 and 2012. We chose these two disciplines as their main difference is rooted in the connectivity between interdisciplinary as well as novel lines of research and established knowledge: While physics is characterized by a strong, paradigmatic core and a large community with the ability to integrate new knowledge easily [37–39], psychology is multi-paradigmatic, diverse, and characterized by different schools of thought [40], which are in epistemological conflict with each other [41–44], making the integration of new knowledge more difficult compared to physics. Consequently, we assume these disciplinary differences to be reflected in the effects of novelty and IDR on the research impact of early career researchers.

To reconstruct the publication history of the ECRs, we merged Web of Science (WoS) data with data on PhD theses from the ProQuest database. Methodologically, we transfer the diversity measures *variety*, *disparity*, and *balance* [15, 45], and *novelty* [46] from the article level to the author level, and employ linear regression models to investigate their association with research impact measured in citations. This author-centric approach is necessary to investigate the influence of interdisciplinarity and novelty on the research impact of ECRs beyond case studies and provides a valuable insight for investigating academic careers.

Our results show that conducting interdisciplinary research as an early career researcher has different ramifications in physics and psychology: While it is beneficial for early career physicists, it is negatively associated with research impact in psychology. Focusing on novel combinations on the other hand is associated with higher impact for early career physicists as well as early career psychologists. Our findings, hence, underline that ECR who deviate from mainstream research in their early years as a scholar might profit by gaining a “recognition premium”.

## Related work

### Interdisciplinarity and novelty

IDR is defined as research that draws from topics, research methods, theoretical approaches, and empirical methods situated in different disciplinary domains such as physics and chemistry [47, 48], whereas novelty implies the (re-)combination of existing topics in unusual ways [14, 49]. Different from that, the first appearance of new concepts, theories, or methods is often coined originality [19]. As we seek to translate and test the approach on novelty taken on the article level, which focuses on recombinant novelty, we refrain from applying originality in the article at hand.

It is important to note, that novelty and IDR are not the same [19, 50, 51]. Research in solid state physics or condensed matter physics, for instance, overlap with materials science, which in turn combines the two fields of physics with chemistry and engineering. Albeit some research questions, techniques, and methods might be novel developments, combining solid state physics/condensed matter physics with chemistry or engineering are not. The same applies for cognitive sciences, which incorporated parts of psychology in the 1970s and linked them with biology, linguistics, and computer science [52]. Again, research areas like cognitive psychology may take insights from the cognitive sciences and thus are interdisciplinary, yet this combination might not yield novel lines of research.

Given the “high risk, high gain” [14] nature of IDR, it is unsurprising that previous research found mixed results on its association with research impact. On the one hand, IDR plays an important role in the emergence of high impact research [13, 15, 53], although at the cost of productivity [20]. On the other hand, IDR is found to be detrimental for research impact. This is attributed to the considerable hurdles stemming from epistemological conflicts [54] and opportunity costs arising from the coordination between scholars and knowledge stemming from different disciplines [18, 55].

Similar to IDR, previous studies provide mixed evidence on the association between novelty and research impact as well as publication output, depending on the combination of novelty and conventionality [12, 14]. Besides an outright “bias against novelty” [56] the main drawback of publishing articles addressing novel lines of research is the often late appreciation of such work in terms of citations [57]. If novel work gets published, however, it is expected to garner more citations over time than less integrative work [46], especially if it is published in high impact journals [58, 59].

In summary, previous studies suggest interdisciplinary research and the pursuit of novel lines of research to only pay off under specific circumstances. Research with high novelty value is acknowledged only belatedly (if at all) which poses a problem for early career researchers, as they have to demonstrate that their research is being acknowledged by their peers within a short time window. IDR on the other hand requires higher cognitive and organizational investment to coordinate researchers and knowledge.

### Early career

Previous studies emphasize the importance of research output for ECRs and simultaneously highlight the risks associated with pursuing novel and interdisciplinary lines of research. For example, Nicholas et al. [29] conducted 116 in-depth interviews with ECRs from different countries, showing that their performance is judged primarily by the number of papers published, the impact factors of the journals in which the research is published, and the receipt of grants and awards. This importance of citations, publications, and impact factors of the outlets for early career scholars is widely supported [30–32]. In contrast, other factors, such as the pursuit of novel lines of research or interdisciplinarity, have not been investigated as relevant performance indicators for ECRs.

Marcella et al. [33] also emphasize that research impact (measured traditionally in terms of citation counts as well as in terms of outreach) is the most relevant currency of ECRs and mid-career researchers. Interviewees also addressed the need to adapt their research methods and their choice in topics in order to maximize their impact scores. Laudel and Gläser [25] provide evidence from a pilot study for negative effects of following new lines of research as ECRs “require an extended learning period to acquire knowledge about a new area immediately after the PhD” [25]. ECRs that are interested in interdisciplinarity, expect negative prospects for their academic career if they pursue interdisciplinary work [34, 35].

Holley [36] as well as Yoshioka-Kobayashi and Shibayama [21] argue however that ECRs, which are encouraged to deviate from conventional research topics, tend to achieve greater cognitive independence and produce more original research output. Nonetheless, scientists associate IDR with applied research and a lack of scientific rigor, making it more difficult for early career researchers to acquire the merits needed for tenure [60, 61].

### Rationale for comparing physics and psychology

We compare early career physicists and psychologists for three reasons: Firstly, we want to investigate if the association between novelty and IDR on the one hand, and research impact on the other hand, differs in general between natural sciences and behavioral sciences. Secondly, both disciplines are key examples for the strength/weakness of a field’s paradigmatic core [62]. Physics relies on a common core revolving around mathematical expressions of natural phenomena, experimentation, and a close dialogue between theory and experiments to provide predictions. This allows scholars to follow novel, interdisciplinary lines of research such as quantum computing [63] or social physics [64, 65]. Psychology is much more fragmented and relies on many theories, methodological approaches, and schools of thought like behaviorism or psychoanalysis [40]. There is also still a debate on the very subject of psychology (the mind, behavior, psyche) and whether it can be unified under a single paradigm [41–44]. Because of the fragmentation of the field of psychology, researchers need to signal their belonging to a school of thought which lowers the chances to get acknowledged for deviant lines of research. Finally, physics and psychology differ in the fact that the knowledge of the former discipline can be translated into technologies and often leads to applied, interdisciplinary research (e.g., with material sciences, biotechnology) [66]. Accordingly, in physics, interdisciplinary research is expected to be more beneficial for ECRs than in psychology.

In sum, pursuing novel lines of research, having a broad research portfolio, and pursuing interdisciplinary research appear to be detrimental for the scientific success of ECRs. Because of the differences between fields, in our case between physics and psychology, these effects are expected to differ in strength and even direction.

## Data and methods

### Data collection and preparation

To answer our research question, we compiled a unique dataset that combines bibliographic, biographic, and institutional data. We gathered information on citation counts, and disciplinary classification of the outlets for bibliographic information from WoS. In the next step, we applied a name disambiguation routine, and assigned aggregated publication counts, citation counts, and disciplinary classifications to the ECRs under investigation. More detailed information on the construction of the dataset is provided in S1 File.

We combined the WoS author data with ProQuest data. ProQuest provides us with information on the PhD theses, namely institutional affiliation and year of publication. We used this information to reconstruct the start of the career and alma mater prestige. Combined, the WoS and ProQuest data allow us to reconstruct the (early) careers of physicists and psychologists who published their dissertation between 2008 and 2012. We consider researchers if they published at least two articles after the publication of their dissertation. This way, we are able to compute our measures but we also exclude PhDs who, most likely, do not consider to pursue a scientific career. We cannot distinguish between ECRs who stay in academia for a longer time from those leaving the academic field after our, still relatively short, observation period.

In contrast to most previous studies, we operationalize our dependent and independent variables on the author level. This allows us to focus on early careers, rather than on single papers. Using researchers’ publication records so enables us to trace whether ECRs direct their attention to interdisciplinary research rather than, for example, just publishing a single article that could be considered as IDR. In so doing, we can address whether the higher cognitive and organizational investment mentioned above influences their overall impact. Simply put, we want to investigate the aggregated effect of research strategies applied by early career researchers across papers on their aggregated impact.

The early career period is defined as the first six years after receiving a PhD degree. We provide model comparisons for different career lengths in S2 File to compare the somewhat arbitrary choices for what constitutes the early career. The comparison shows no substantial differences. Because of the multiple constraints on considered researchers (career start, length of early and established career, missing data etc.), we end up with a sample consisting of 4003 physicists and 4097 psychologists who graduated at US universities between 2008 and 2012.

### Operationalization

#### Dependent variable

**Fig 1:**
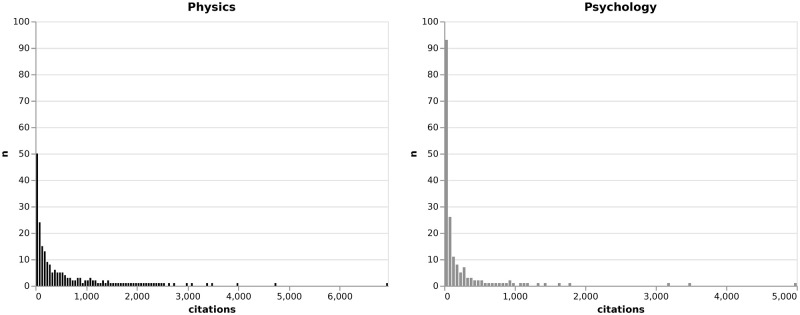
Citation distributions. Number of absolute citations for physics and psychology, respectively.

In line with previous studies, we use the accumulated citation count as a measure of research impact [13, 14, 19, 20, 46, 67]. The more a researcher gets cited, the more they are recognized by other scientists. For every article an author published, we add the citations it received in the first three years after its publication. We use a three-year citation window to have a comparable citation measure and to include as many articles and authors as possible. A larger citation window, especially for more than five years would shrink our sample substantially. To be sure, we provide robustness tests for a longer citation window in S3 File. As is the nature of the academic prestige economy, many researchers have a low mean impact and few researchers have a high impact [68]. Therefore, the mean citation distribution is heavily skewed (cf. Fig 1) and we use logged citation counts in the regressions.

#### Explanatory variables

As discussed above, novelty measures the degree to which an author employs innovative combinations of topics, whereas IDR focuses on disciplinary-spanning behavior. From an empirical point of view, interdisciplinarity is a multidimensional concept and therefore commonly subdivided into *variety*, *balance* and *disparity* [15, 45, 69]. For example, a researcher might routinely address only a few subjects (low *variety*) but combines intellectually disparate subjects such as geriatric and sports psychology (high *disparity*). Another scientist might pursue the opposite strategy and look into many different subjects that are intellectually close and commonly combined.

**Fig 2:**
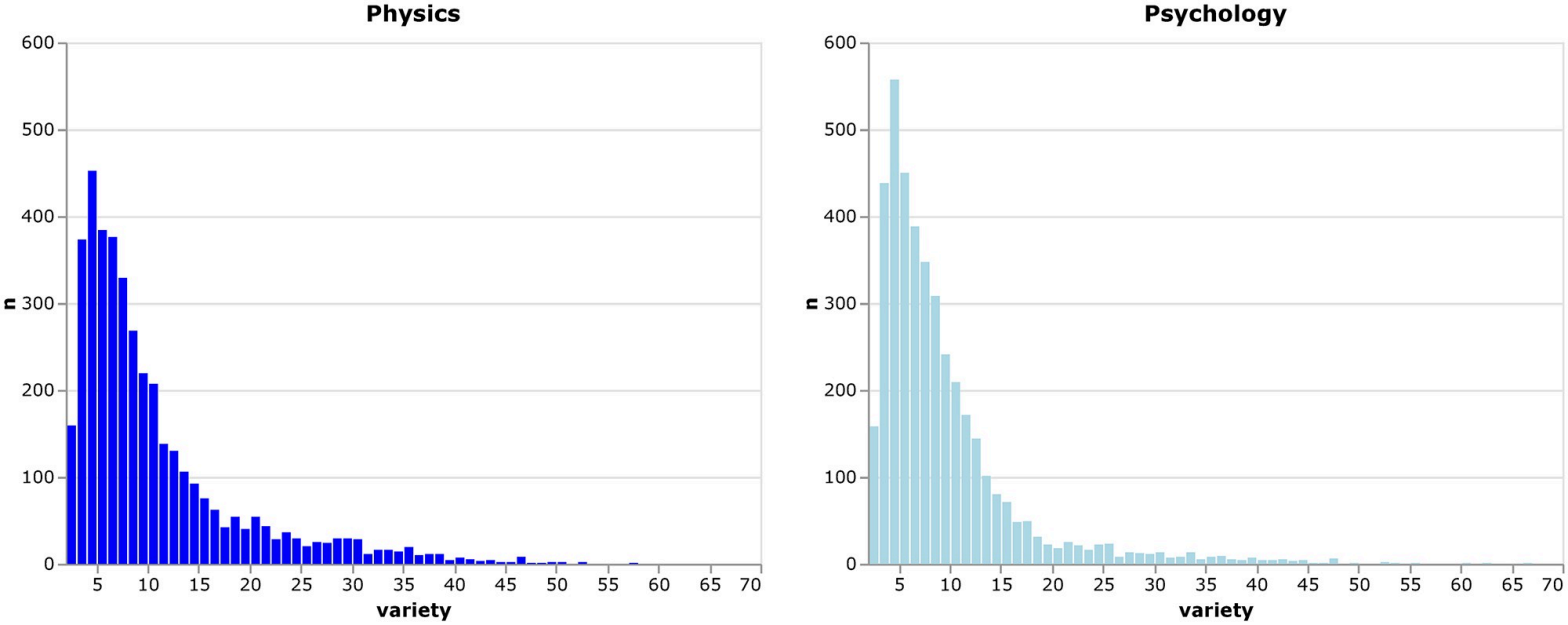
Distribution of variety. Distribution of the *variety* of early career researchers’ publications in physics and psychology, respectively.

We use journal classifications provided by WoS to construct our indicators of interdisciplinarity and novelty [15, 45, 46]. While the use of journal classifications is a simplification [70], they are widely employed for research on individual articles and we therefore use them to showcase how to use this approach on early careers. As WoS provides at least one classification for every indexed outlet [71], we are able to assign journal classifications to the articles and to aggregate them for ECRs. We operationalize our key explanatory variables *variety, balance, disparity*, and *novelty* as follows:

*Variety* measures *how many different subjects are combined* in a single paper, or in our case, by a single author. A high *variety* would require at least a partial understanding of many different subjects, and thus increases the cognitive burden on early career researchers as outlined above. *Variety* is typically defined as the number of distinct journal classifications (*n*_*c*_) among cited sources of a given article [15]. To obtain the *variety* on the author level, we count the distinct journal classifications of an authors articles (*n*_*r*_). Fig 2 shows that the *variety* distribution is heavily skewed to the right, which means that most ECRs in our sample publish only within few different subjects.

**Fig 3:**
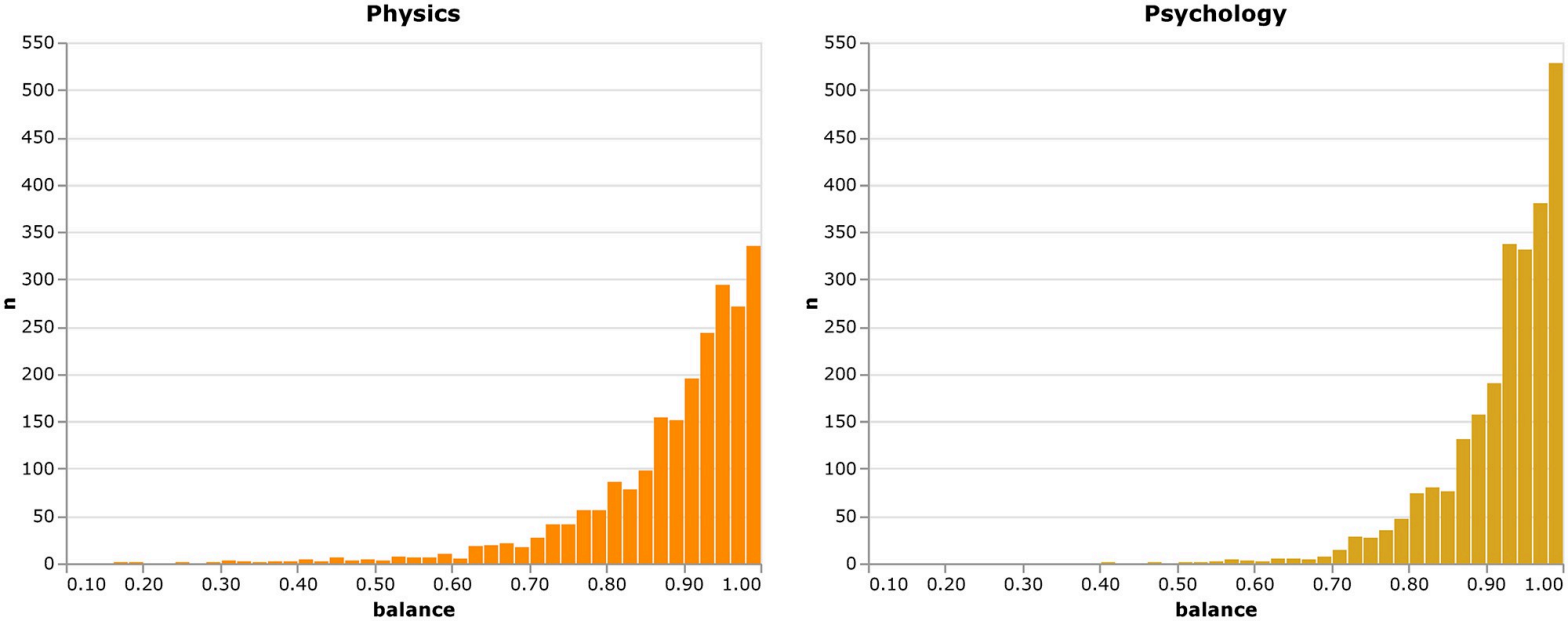
Distribution of balance. Distribution of the *balance* of early career researchers’ publications in physics and psychology, respectively.

*Balance* gauges the evenness of the subject distribution. Low values of balance indicate a narrow focus on a limited field of study as a strategy for achieving higher levels of research impact. While a low *variety* of, for example, two subjects could also be seen as a narrower focus, it is still possible that a researcher addressed subject *A* once and subject *B* ten times. A high *balance* on the other hand requires rather equal attention to all addressed subjects. We employ the modified Shannon-Diversity to capture *balance* as a second dimension of interdisciplinarity [15]. Balance is formalized as:

$$\begin{array}{c} balance=-\frac{1}{ln(n_{r})}\sum_{i}p_{i}\cdot ln\left(p_{i}\right) \end{array}$$ (1)

where *n*_*r*_ denotes the aforementioned *variety*, and *p*_*i*_ is the proportion of an authors publications with WoS category *i*. The distribution of *balance* is heavily skewed to the left as can be seen in Fig 3. This means, that most ECRs in our sample either publish articles in few subjects or very evenly across subjects.

**Fig 4:**
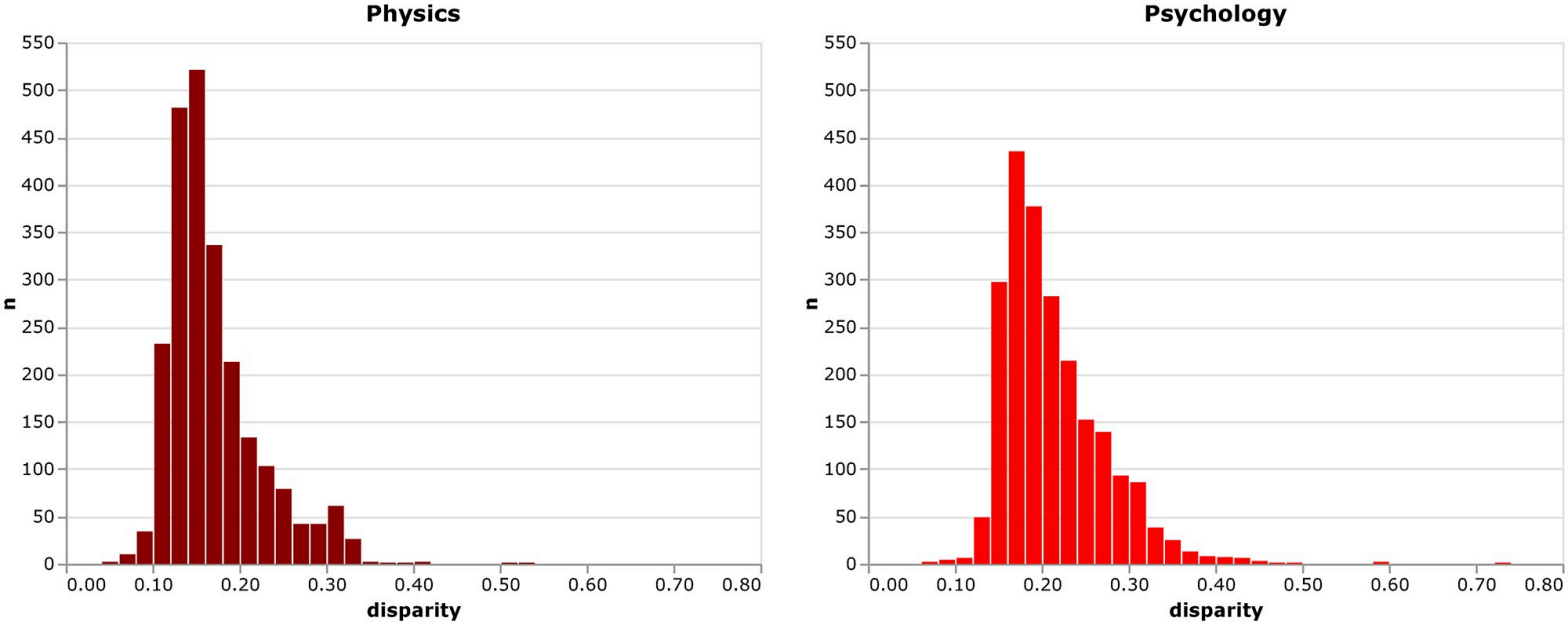
Distribution of disparity. Distribution of the *disparity* of early career researchers’ publications in physics and psychology, respectively.

The final diversity metric, *disparity*, measures the cognitive distance between the combined subjects. This measure is applied to answer the question of *how different the subjects* are [45], which are addressed by ECRs. Due to the analysis of early career researchers instead of research papers, we modify *disparity* introduced by Yegros-Yegros et al. [15] as follows: First, we create a *n*×*m* matrix for every year in our dataset where *m* is the number of classifications and *n* is the number of ECRs who started their career in or before that year. Every row (representing an ECR) in this matrix contains 1 if the corresponding ECR has published any article that is tagged with the associated classification, and 0 otherwise. Only classifications on items that are published during the early career time window are included. From that, we construct a co-occurrence matrix that indicates how often any two classifications are present within the ECRs in our dataset. To calculate the *disparity* measure on the author level, we extract every combination of classifications that is present within that author. For each combination, we calculate the cosine similarity based on the column vectors of the coocurrence matrix and divide the sum of all similarities by the number of combinations. This procedure can be formalized as:

$$\begin{array}{c} d_{ij}=1-s_{ij} \end{array}$$ (2)

where *s*_*ij*_ is the cosine similarity between two journal classifications *i* and *j*, in other words their vectors in the co-occurrence matrix. The cosine distances are then averaged for a given author, resulting in the authors’ *disparity* measure,

$$\begin{array}{c} disparity=\frac{1}{n_{r}\left(n_{r}-1\right)}\sum_{ij}d_{ij} \end{array}$$ (3)

where *n*_*r*_ is *variety* as described above. From the distribution of *disparity*, shown in Fig 4, it becomes apparent, that neither the physics ECRs nor the psychology ECRs have extreme disparity values. The distribution of *disparity* is not as heavily skewed as *variety* or *balance*, but many of the ECRs in our sample combine less distant (sub-)disciplines.

To further highlight why we discuss the measures above separately, we provide the correlation matrices of all variables used in the model for both disciplines in Tables 1 and 2. As can be seen, the three diversity metrics have low correlations with each other, each revealing a different aspect of IDR [15].

**Table 1:** Physics correlations.

	Gender	Elite	N(Articles)	Variety	Balance	Disparity	Novelty
Gender							
Elite	0.02						
N(Articles)	-0.01	0.03					
Variety	0.02	-0.03	0.70***				
Balance	0.01	-0.07***	-0.44***	0.02			
Disparity	-0.02	0.10***	0.01	-0.21***	-0.25***		
Novelty	-0.02	-0.04*	-0.09***	-0.04*	0.09***	-0.27***	
N(Citations)	-0.01	0.12***	0.63***	0.24***	-0.44***	0.12***	-0.06***

***p < 0.001;

**p < 0.01;

*p < 0.05

Correlations for the physics sample. Own Calculations.

**Table 2:** Psychology correlations.

	Gender	Elite	N(Articles)	Variety	Balance	Disparity	Novelty
Gender							
Elite	-0.02						
N(Articles)	-0.09***	0.07***					
Variety	-0.06***	0.05***	0.84***				
Balance	0.06***	-0.03	-0.39***	-0.07***			
Disparity	-0.12***	0.00	0.07***	0.14***	0.06***		
Novelty	0.05***	-0.01	-0.13***	-0.13***	0.02	-0.31***	
N(Citations)	-0.06***	0.08***	0.62***	0.52***	-0.21***	0.01	-0.07***

***p < 0.001;

**p < 0.01;

*p < 0.05

Correlations for the psychology sample. Own Calculations.

While *disparity* compares an author’s publications to their early career peers, *novelty* compares the disciplinary combinations against publications of early career researchers who entered the market before them to explore how new their combination of subjects is. Implementations of the novelty measure range from having two articles that have been cited together before [19] to more complex approaches looking at journal classifications in referenced works [14, 46].

**Fig 5:**
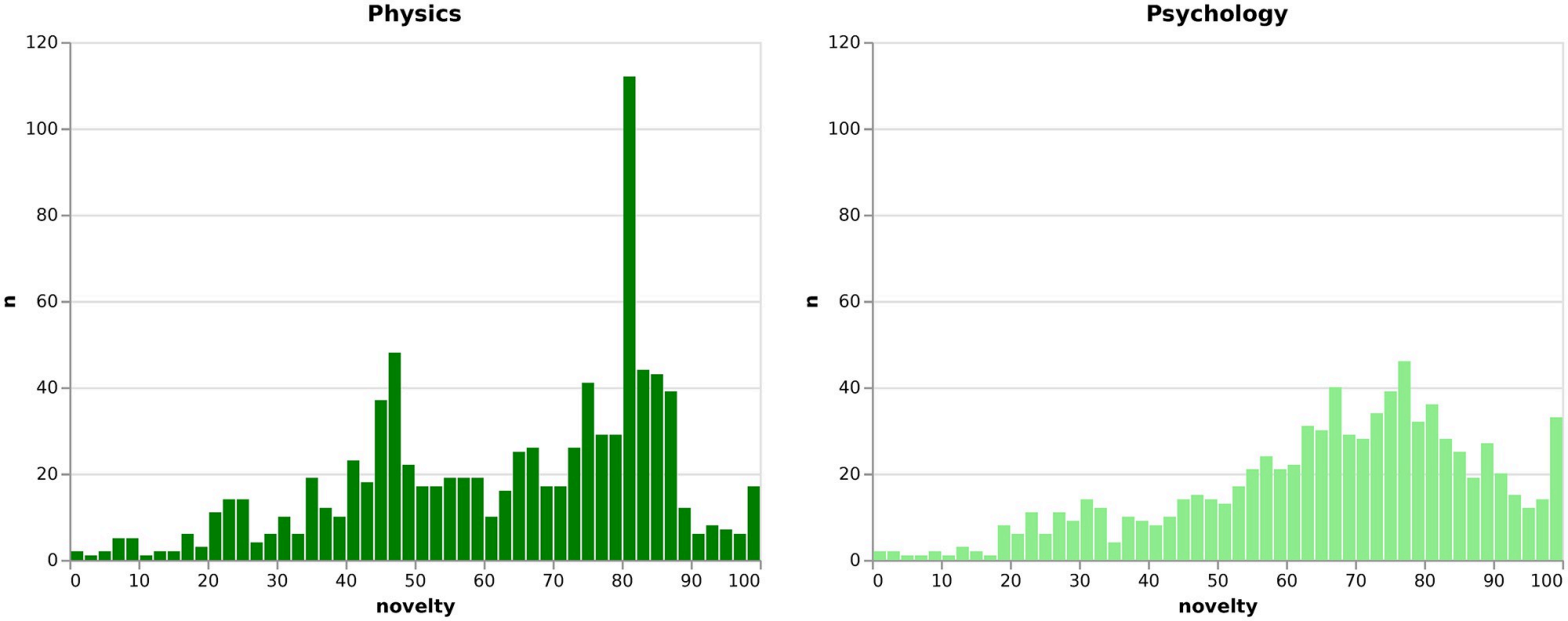
Novelty distribution. Distribution of the independent variable *novelty* for the physics and psychology sample.

We modify the approach suggested by Leahey and Moody [46] and compare a given article to all other articles in our sample that were published up to three years prior, and only within the same discipline. We use three years instead of Leahy and Moody’s [46] five years to have a larger sample at our disposal but supply a comparison to smaller sample with a five year *novelty*-range in S4 File that shows no major discrepancies to our main model below. Note that we focus on combinatorial novelty, which is concerned with combinations of classifications that already exist [19]. Discoveries that are novel but do not combine previous concepts, methods, etc. are not picked up by this measure and are out of scope for this article. For a given combination of subjects (e.g., high energy physics, quantum mechanics), novelty measures the ratio of articles published with this specific combination to all articles issued in both subjects. We again look at the classifications assigned to articles and compare the number of expected articles with a given combination *N*_*expected*_ (see formula 4) to the observed number of articles with the combination *N*_*observed*_ for each classification:

$$\begin{array}{c} N_{expected_{ij}}=\frac{c_{i}c_{j}}{N} \end{array}$$ (4)

$$\begin{array}{c} novelty=1-\frac{N_{observed}}{N_{expected}} \end{array}$$ (5)

where *c*_*i*_;*c*_*j*_ are the numbers of articles with at least classification *i*;*j* and *N* is the number of all articles. We express novelty as percentiles for easier interpretation of the resulting scores [46]. Because an article can have more than one classification combination, we assign each article the highest percentile score of all its classification combinations i.e. the most novel score, while an author is assigned the mean of all their articles novelty measures. Fig 5 shows that the *novelty* distribution is slightly left skewed for the ECRs in our sample.

As seen in Tables 1 and 2, *novelty* and *disparity* are negatively correlated. This correlation mirrors the differentiation of IDR and novelty as introduced earlier. It indicates that a combination between disparate areas of research might actually not be as novel as initially thought, e.g. social physics, which was already discussed in the 1950s [72, 73], or research in materials science [74] or cognitive sciences [52].

#### Control variables

We include graduation from an elite department, gender, year of career start and the number of articles published as control variables in our models. We define *graduation from an elite department* as a dichotomous variable with 1 = author graduated from an elite department and 0 otherwise. Elite departments are defined as the ten highest ranked U.S. universities in physics and psychology respectively, according to the U.S. News & World Report rankings [75, 76], see S6 File for further information. The genderize service was employed to assign *gender* to the respective early career scientists [77, 78]. We used first names and countrycodes (where provided) as information for assignment and denoted 1 for female and 0 for male early career scholars. At last, *career start* was defined as the year in which a scientist’s dissertation was published. This way we control for changes over time, for example publication practices within a discipline.

### Regression model

We calculated a linear regression model with log(*Impact* + 1) as dependent variable [13, 15]. Robust standard errors are employed since the residuals are heteroskedastic for both samples as indicated by Breusch-Pagan (BP) tests. We furthermore calculated the generalized variance inflation factor (GVIF) to test for multicollinearity. According to our test results, multicollinearity is not present in the models. Results for the BP tests and GVIF are provided in S7 File. We also compare our results from the log-transformed linear regression model to a negative binomial regression model, which can also be used to model citations as a dependent variable [12, 53, 56]. The results are similar and are discussed in S8 File.

## Results

**Fig 6:**
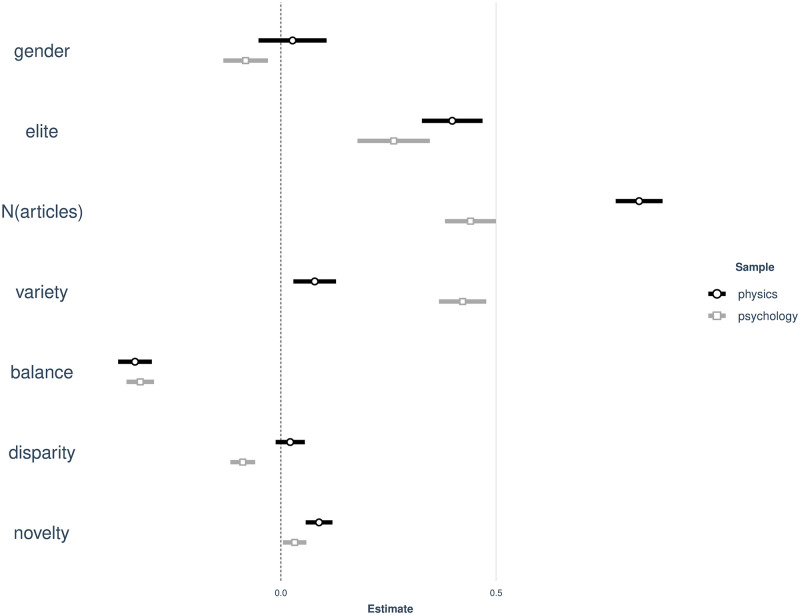
Main effects of regression. Main effects for physics and psychology (95% confidence intervals).

Our results reveal a number of similarities, but also important differences between physics and psychology. As can be seen in Fig 6 differences arise in three instances: Firstly, in regards to *disparity* as a dimension of IDR. This notable difference manifests in a highly significant, negative association between disparity and research impact for ECRs in psychology, meaning that addressing distant subjects is detrimental to ECRs in psychology. The citation count of ECRs in physics is however not affected by combining highly disparate areas of research. Secondly, the effect size of *novelty* for physics ECRs is considerably smaller compared to physics and near zero. Researching new combinations is therefore more beneficial for ECR physicists. Finally, we see differences in the *gender* effect, which is negative for ECRs in psychology and nonexistent in the case of physics.

More detailed results are presented in Table 3. Regarding the other dimensions of IDR, our models reveals that *variety* is positively associated with research impact of ECRs. Publishing in multiple different subjects, exerts a more positive, highly significant effect in psychology compared to physics. This finding suggests that keeping focused on one particular topic is relevant for early career physicists to generate research impact, while ECRs in psychology have to cover a wider range of topics to get cited. However, the findings indicate that the latter should still focus on a limited number of main lines of research, as our *balance* measure further suggests. The same holds true for physics, as there is a similar effect for physics as indicated by both the effect plot (Fig 6) and Table 3. Regarding *novelty*, our models reveal small, positive associations with research impact of ECRs for both samples. In case of physics, *balance* yields the strongest effect size among the main explanatory variables. This effect is overshadowed by a larger positive effect of *variety* in the case of psychology. This suggests that a certain degree of specialization is needed and must be transferred to other subfields of psychology to be acknowledged by peers. At the same time, the expertise associated with this specialization should not be transferred to proximate topics as indicated by the negative values of the *disparity*-measure.

**Table 3:** Results of the linear regressions on the logged citation count.

	physics sample	psychology sample
(Intercept)	4.37*** (0.04)	3.52*** (0.03)
Gender	0.03 (0.04)	−0.08** (0.03)
Elite	0.40*** (0.04)	0.26*** (0.04)
N(articles)	0.81*** (0.03)	0.42*** (0.04)
Variety	0.08** (0.02)	0.41*** (0.03)
Balance	−0.34*** (0.02)	−0.33*** (0.02)
Disparity	0.02 (0.02)	−0.11*** (0.02)
Novelty	0.09*** (0.02)	0.03* (0.02)
Years fixed	yes	yes
Adj. R^2^	0.56	0.58
AIC	11026.22	10081.78
Log. Lik.	−5500.11	−5027.89
Num. Obs.	4003	4097

***p < 0.001;

**p < 0.01;

*p < 0.05

Results of the linear regressions on ECRs research impact. All non-dummy explanatory variables have been standardized before modeling.

As for our control variables, we find that *graduation from an elite department* entails more citations compared to having graduated from a less prestigious department. This effect is stronger for physics compared to psychology. Among all variables included in the models, the effect of the number of articles published is the strongest and shows that productivity pays off in citation counts. However, this finding should be read with caution, as working in larger teams, especially being part of a collaboration in physics, yields a premium on citation counts. From the interaction plots presented in S9 File and the correlations in S10 File it is clear, that the effects of *variety* and *balance* rely heavily on the number of articles published. *Variety* is also not robust for different career length operationalizations which is shown in S2.1 and S2.3 Table in S2 File.

Nevertheless, our models suggest that even when controlled for publication count, affiliation with elite departments, gender, IDR measures and novelty have a substantial and nuanced impact on the citation count of ECRs.

## Discussion and conclusion

In this article, we studied if and how interdisciplinarity and novelty affect the research impact of ECRs in psychology and physics. Both strategies are key to the dynamics of science, to what research is getting favored, and therefore of high political interest. If the trade-off for young scientists swings towards more traditional paths and disciplinary boundaries, it counteracts prominent ideas of “big jumps” often associated with innovative technological solutions.

To this end, we reconstructed the publication record of 4003 early career physicists and 4097 early career psychologists who graduated between 2008 and 2012 at U.S. universities. We followed Yegros-Yegros et al. [15] in subdividing IDR into a multidimensional concept composed of *variety*, *balance*, and *disparity*, and Leahey and Moody [46] to measure novelty. However, we calculated the metrics on the author-level allowing us to consider a career perspective and test the impact of IDR and novelty on young scientists’ research impact.

We expected that IDR yields higher returns in terms of research impact for physics ECRs, while lowering the impact of ECRs in psychology. We attributed this to disciplinary differences, namely the common paradigmatic core in physics [66] and the internal fragmentation and multiparadigmatic constitution of psychology [43, 44, 79]. While the former supposedly enables ECRs to pursue IDR as long as it is connectable to the common paradigmatic core, the latter requires ECRs to signal their belonging to a school of thought, thus limiting their possibilities to gain recognition from conducting IDR. Additionally, we expected a negative association between the pursuit of novel lines of research and research impact for ECRs in both disciplines according to the literature [19, 20]. We find that addressing many different subjects, as measured by *variety*, is beneficial for both early career physicists and psychologists. This effect is much more pronounced for psychology, and indicates that ECRs in psychology profit from spanning multiple subjects in order to grow their impact, measured in citations.

Regarding the different facets of IDR, we uncover a nuanced picture with differences between the impact of interdisciplinarity and novelty on psychology and physics ECRs. According to the strong effect of the *balance* measure in both samples, different subjects should however not be addressed with equal dedication pointing to the success of a certain degree of specialization both for physics and psychology ECRs [80]. A balanced research portfolio might signal that ECRs cannot decide on a line of research and thus at not having developed a research agenda on their own [10, 27], or that the ECR pursues research that does not fit into the general discussion of either physics or psychology and it is therefore more difficult to evaluate whether it meets the standards of the respective discipline [60, 61].

As indicated by the differences in the *disparity*-measures, psychology appears more fragmented than physics, giving an advantage in citation counts to those who combine research topics in close proximity. The negative effect of *disparity* present for ECRs in psychology hints at epistemological conflicts between different paradigms as seen in previous papers on the epistemological foundation of psychology [41–44]. In fact, bridging different paradigms might cost the connectivity to established discourses of an ECR in the addressed (sub-)disciplines.

Bridging disparate areas of research is neither beneficial nor detrimental for ECR physicists, but covering a large variety of topics does not yield the same benefits as in psychology. This might be the consequence of the high specialization pressure in physics as well as the higher levels of embeddedness of ECRs in laboratories and research collaborations [81, 82]. Within these structures, physicists might do well to find their niche within already highly specialized research areas as there might be simply no room for developing a research agenda that spans highly disparate topics. Yet, if ECRs in physics do so, they might be able to claim their position within the discourse of physics, as long as they refer to the common, paradigmatic ground. This may be grounded in the larger integrative power in physics, allowing unusual topic combinations (e.g., quantum dynamics).

Our findings imply that following IDR lines of research in close intellectual proximity might help to improve the intellectual prominence of ECRs in line with [11], but is linked to considerable intellectual strain if different lines of research are followed equally. Our results also indicate considerable opportunity costs involved for ECRs in both disciplines, which was also found for individual articles [17, 18, 55]. Yet, the hurdles stemming from epistemological conflicts [20, 54] are stronger for the behavioral sciences like psychology compared to physics. This may hold true for disciplines in the behavioral sciences and social sciences in close proximity to psychology [83, 84].

As indicated above, the impact of IDR takes different forms in regards to the strength of the paradigmatic core of the discipline under investigation. Our regression results show that a higher overall topic diversity does not necessarily result in higher citation counts for early career physicists. For psychology however, we observe an overall negative effect of IDR on citation count for early career researchers. Thus, ECRs who are able to choose between different “high risk, high gain” [14] strategies, have to consider the nature of their field in order to optimize their research impact.

The pursuit of novel lines of research as indicated by the *novelty* metric contradicts our expectations to a certain extent. In both samples, we see small, but positive associations between novelty and research impact. At least for early career physicists and psychologists, addressing novel lines of research pays off quickly enough when looking at their whole (early) work, even though it might still be risky for individual papers. The requirements for ECRs to develop an independent research identity and to contribute novel insights to the scholarly discourse as part of their PhD and postdoctoral studies might be more important than the drawbacks, which is also supported by recent results for young researchers in the sociological field [6].

There are noticeable differences for ECRs based on departmental prestige to accumulate a large number of citations. This is indicated by the strong, positive effect of having graduated from an elite-department and is in line with the transmission of prestige and visibility [28]. This could also indicate more favorable working conditions at elite departments, like more research autonomy which may translate to innovative research and ultimately more citations.

Our study has a number of limitations, which are mostly related to our data. Firstly, we are unable to consider the dropout of students prior to completion of a PhD. The zero effect for gender in physics, for example, may be caused either by the fact that few women self-selected into the program or dropped out at the time they obtained their PhD. We base our analysis on WoS-data for reasons of data availability. There are however more comprehensive databases available from which future studies should draw (e.g., PsycInfo for psychology). Secondly, we base our measures upon journal classifications, which are assigned to articles and finally aggregated on the author level. On the one hand, the simplicity of the journal classifications is very helpful as it makes the operationalization easier, on the other hand it can be argued, that it is too simple to capture the full breadth of the content of articles. This also reflects on the fact that we we can only consider combinatorial novelty. Thirdly, different fields might have different classifications schemes which could make comparisons less accurate. Further research in this direction is needed, for example by using topic models or other natural language processing tools on the content of articles. To investigate the interdisciplinarity of individual articles, the measures are applied to classifications of cited sources instead of classifications of the article itself, which leads to a higher number of classifications. Our career approach however takes only classifications of articles written by an individual author into account which provides an overall lower number of classifications. We fourthly restricted our career window to six years after obtaining a PhD, which is a compromise between the number of classifications that are available and the size of our sample. Longer durations after the beginning of scientific careers would allow more in-depth analyses of the timing of interdisciplinarity within careers and we encourage further research in this direction. Future studies should therefore either look into applying our career approach to full careers or apply it to classifications of cited sources of articles written by the researchers. This way career effects can be investigated further and future studies could take advantage of a higher number of classifications, mitigating the dependency of *variety* and *balance* on the number of articles published. To obtain the necessary data might, however, be a challenge in and of itself. Finally, we restricted our sample to early career physicists and psychologists who graduated between 2008 and 2012 at U.S. universities. We are therefore limited in the generalizability of our findings. For example, other countries (e.g., Germany with its chairholder system) and other mono- and multiparadigmatic disciplines might reveal different associations between IDR, novelty, and research impact for ECRs.

Despite these limitations, our findings indicate that early specialization, lower novelty, high numbers of articles published, as well as gaining a PhD from an elite department are relevant to get the research impact necessary for ECRs to survive in the competitive environment of academia. Furthermore, we encourage scholars who wish to replicate our findings to use different databases, for example PsycInfo or Scopus. By doing so, they might not only provide information on the validity of our findings, but also on possible biases present in the WoS database.

On a final note: should ECRs pursue novel or interdisciplinary lines of research following our results? Our findings imply that ECRs should pursue a limited number of research topics and make one their main line of research [6]. In addition, ECRs should avoid trying to integrate intellectually disparate research topics. Finally, our results may encourage young scholars to pursuit novel thematic combinations as a potential strategy to gain recognition from other scientists.

## Supporting information

S1 File Data disambiguation. (PDF)

S2 File Robustness Check I: Variation in early career length. (PDF)

S3 File Robustness Check II: Varying citation window. (PDF)

S4 File Robustness Check III: Variation in novelty range. (PDF)

S5 File Simple examples for all explanatory variables. (PDF)

S6 File List of elite universities. (PDF)

S7 File Regression diagnostics. (PDF)

S8 File Robustness Check IV: Negative binomial regression model. (PDF)

S9 File Robustness check V: Interaction between the main effects. (PDF)

S10 File Descriptives. (PDF)

S11 File Aggregated data: Physics. (CSV)

S12 File Aggregated data: Psychology. (CSV)
