# Supplementary material for: Benefits and detriments of interdisciplinarity on early career scientists’ performance. An author-level approach for U.S. physicists and psychologists
Source: PLoS One. 2022 Jun 30;17(6):e0269991. doi: 10.1371/journal.pone.0269991 (PMC9246137; doi:10.1371/journal.pone.0269991)
Supplement: S1 File — (PDF) [file pone.0269991.s001.pdf]

S1 Data disambiguation

We constructed our sample by selecting scholars who graduated in physics and psychology from the ProQuest (PQ) database. We did so by manually scanning all available user generated PQ-tags and selected a number of directly related tags (see Table 1 for an overview). We then performed a search for each ECR in the Web of Science database. The threshold for the ECRs to remain in our sample was set to the publication of least one scientific article within 3 years before and 5 years after the acquisition of a PhD-degree at their respective university. ECRs who published less were removed from our sample. Only uniquely identified scholars were then added to the final dataset. After our matching procedure we ended up with 32.785 authors (17710 physics, 15075 psychology) of which we kept 17684 (9708 physics, 7976 psychology) due to our career start constraint. As a last step we excluded authors if they had recorded more than twenty articles per year in the years following their graduation, leaving us with 4003 early career physicists and 4097 psychologists. This last step ensures that cases with a high probability of consisting of more than one author are excluded.

Table 1. PQ categories.

psychology	physics
psychotherapy	astrophysics
socialpsychology	biophysics
physiologicalpsychology	solidstatephysics
clinicalpsychology	geophysics
educationalpsychology	particlephysics
psychology	condensedmatterphysics
developmentalpsychology	nuclearphysics
occupationalpsychology	physicalchemistry
counselingpsychology	physics
psychologicaltests	plasmaphysics
behavioralpsychology	lowtemperaturephysics
cognitivepsychology	quantumphysics
quantitativepsychology	geophysical
experimentalpsychology	theoreticalphysics
personalitypsychology	molecularphysics
childpsychology	hightemperaturephysics
psychopathology	
psychologicalaspects	
psychologists	
neuropsychology	
psychoanalysis	
psychotropicdrugs	
geriatricpsychology	
sportspsychology	
schoolpsychologists	

ProQuest user generated keywords by field.
