# Supplementary material for: Benefits and detriments of interdisciplinarity on early career scientists’ performance. An author-level approach for U.S. physicists and psychologists
Source: PLoS One. 2022 Jun 30;17(6):e0269991. doi: 10.1371/journal.pone.0269991 (PMC9246137; doi:10.1371/journal.pone.0269991)
Supplement: S2 File — (PDF) [file pone.0269991.s002.pdf]

S2 Robustness Check I: Variation in early career length

As a first robustness check, we recalculated our regression models and varied the early career duration between two and six years after graduation. The results of our robustness check are provided in tables S2.1 and S2.2. We consider all ECRs in our database who would fall into the respective early career duration, namely two to six years. In contrast, in the models presented in tables S2.3 and S2.4, we consider only ECRs who would fall into the six-year range that we finally considered. The effects are mostly robust across the models. Nonetheless, we witness changes in the effect sizes of *variety* and *balance* for both samples which could stem from the association with the number of articles issued. This effect might get more pronounced, the longer an ECR is able to issue articles. In addition, the effect of *variety* in physics is not robust across different career lengths. This finding indicates that the pursuit of a variety of topics pays of relatively late in the ECR's career and might thus be considered a high risk strategy in the first years after graduation.

Table S2.1. Career length robustness physics.

	2 years	3 years	4 years	5 years	6 years
(Intercept)	3.62***	3.87***	4.08***	4.25***	4.37***
	(0.04)	(0.04)	(0.04)	(0.04)	(0.04)
Gender	−0.00	−0.00	0.00	0.03	0.03
	(0.03)	(0.03)	(0.03)	(0.04)	(0.04)
Elite	0.32***	0.34***	0.38***	0.41***	0.40***
	(0.03)	(0.03)	(0.03)	(0.03)	(0.04)
N(articles)	0.80***	0.81***	0.82***	0.81***	0.81***
	(0.02)	(0.02)	(0.02)	(0.03)	(0.03)
Variety	−0.03	−0.00	0.02	0.05*	0.08**
	(0.02)	(0.02)	(0.02)	(0.02)	(0.02)
Balance	−0.15***	−0.22***	−0.26***	−0.29***	−0.34***
	(0.02)	(0.02)	(0.02)	(0.02)	(0.02)
Disparity	−0.03	0.00	−0.00	0.02	0.02
	(0.02)	(0.02)	(0.02)	(0.02)	(0.02)
Novelty	0.06***	0.08***	0.08***	0.09***	0.09***
	(0.02)	(0.01)	(0.01)	(0.02)	(0.02)
2009	0.03	−0.00	0.01	0.01	−0.00
	(0.05)	(0.05)	(0.05)	(0.05)	(0.05)
2010	0.01	0.02	−0.01	−0.00	−0.01
	(0.06)	(0.05)	(0.05)	(0.05)	(0.05)
2011	0.01	0.04	0.02	0.02	0.01
	(0.05)	(0.05)	(0.05)	(0.05)	(0.05)
2012	0.02	0.05	0.04	0.03	0.02
	(0.06)	(0.05)	(0.05)	(0.05)	(0.05)
2013	0.10	0.13*	0.08	0.08	
	(0.05)	(0.05)	(0.05)	(0.05)	
2014	0.11	0.11*	0.09		
	(0.06)	(0.05)	(0.05)		
2015	0.06	0.09			
	(0.06)	(0.06)			
Adj. R^2	0.46	0.50	0.53	0.55	0.56
Num. Obs.	4835	5576	5314	4720	4003

$^{***}p < 0.001$; $^{**}p < 0.01$; $^{*}p < 0.05$

Linear regression models for the physics sample with different early career lengths. Additional cases result from shorter career periods.

Table S2.2. Career length robustness psychology.

	2 years	3 years	4 years	5 years	6 years
(Intercept)	2.87***	3.11***	3.28***	3.41***	3.52***
	(0.04)	(0.04)	(0.03)	(0.03)	(0.03)
Gender	−0.07**	−0.07**	−0.06**	−0.06*	−0.08**
	(0.03)	(0.02)	(0.02)	(0.02)	(0.03)
Elite	0.20***	0.22***	0.21***	0.24***	0.26***
	(0.04)	(0.04)	(0.04)	(0.04)	(0.04)
N(articles)	0.47***	0.46***	0.47***	0.45***	0.42***
	(0.03)	(0.03)	(0.03)	(0.03)	(0.04)
Variety	0.19***	0.26***	0.31***	0.35***	0.41***
	(0.03)	(0.03)	(0.03)	(0.03)	(0.03)
Balance	−0.16***	−0.21***	−0.26***	−0.29***	−0.33***
	(0.02)	(0.01)	(0.02)	(0.02)	(0.02)
Disparity	−0.15***	−0.12***	−0.11***	−0.11***	−0.11***
	(0.02)	(0.02)	(0.02)	(0.02)	(0.02)
Novelty	−0.01	0.03*	0.03*	0.04**	0.03*
	(0.02)	(0.01)	(0.01)	(0.01)	(0.02)
2009	0.03	−0.03	−0.06	−0.04	−0.01
	(0.05)	(0.05)	(0.04)	(0.04)	(0.04)
2010	0.03	−0.03	−0.06	−0.07	−0.06
	(0.05)	(0.05)	(0.04)	(0.04)	(0.04)
2011	−0.04	−0.05	−0.07	−0.06	−0.03
	(0.05)	(0.05)	(0.04)	(0.04)	(0.04)
2012	0.02	−0.02	−0.05	−0.07	−0.03
	(0.05)	(0.05)	(0.04)	(0.04)	(0.04)
2013	−0.11*	−0.09*	−0.09*	−0.08	
	(0.05)	(0.05)	(0.04)	(0.04)	
2014	−0.12*	−0.13**	−0.11*		
	(0.05)	(0.05)	(0.05)		
2015	−0.17**	−0.15**			
	(0.05)	(0.05)			
Adj. R^2	0.48	0.51	0.54	0.56	0.58
Num. Obs.	3660	4920	5102	4718	4097

$^{***}p < 0.001$; $^{**}p < 0.01$; $^{*}p < 0.05$

Linear regression models for the psychology sample with different early career lengths. Additional cases result from shorter career periods.

Table S2.3. Career length robustness physics.

	2 years	3 years	4 years	5 years	6 years
(Intercept)	3.62***	3.86***	4.08***	4.25***	4.37***
	(0.04)	(0.04)	(0.04)	(0.04)	(0.04)
Gender	0.03	0.03	0.03	0.03	0.03
	(0.04)	(0.04)	(0.04)	(0.04)	(0.04)
Elite	0.29***	0.33***	0.37***	0.39***	0.40***
	(0.04)	(0.04)	(0.04)	(0.04)	(0.04)
N(articles)	0.76***	0.75***	0.78***	0.80***	0.81***
	(0.03)	(0.03)	(0.03)	(0.03)	(0.03)
Variety	−0.01	0.02	0.04	0.05*	0.08**
	(0.02)	(0.02)	(0.02)	(0.02)	(0.02)
Balance	−0.18***	−0.26***	−0.29***	−0.31***	−0.34***
	(0.02)	(0.02)	(0.02)	(0.02)	(0.02)
Disparity	−0.04	0.01	−0.00	0.02	0.02
	(0.03)	(0.02)	(0.03)	(0.02)	(0.02)
Novelty	0.04	0.07***	0.08***	0.08***	0.09***
	(0.02)	(0.02)	(0.02)	(0.02)	(0.02)
2009	0.03	0.00	0.02	0.01	−0.00
	(0.05)	(0.05)	(0.05)	(0.05)	(0.05)
2010	0.01	0.02	−0.01	−0.00	−0.01
	(0.06)	(0.05)	(0.05)	(0.05)	(0.05)
2011	0.01	0.05	0.02	0.02	0.01
	(0.05)	(0.05)	(0.05)	(0.05)	(0.05)
2012	0.02	0.06	0.04	0.02	0.02
	(0.06)	(0.05)	(0.05)	(0.05)	(0.05)
Adj. R^2	0.45	0.49	0.53	0.55	0.56
Num. Obs.	3030	3513	3758	3914	4003

$^{***}p < 0.001$; $^{**}p < 0.01$; $^{*}p < 0.05$

Linear regression models for the physics sample with different early career lengths. Additional cases are suppressed.

Table S2.4. Career length robustness psychology.

	2 years	3 years	4 years	5 years	6 years
(Intercept)	2.87***	3.11***	3.28***	3.42***	3.52***
	(0.04)	(0.04)	(0.04)	(0.03)	(0.03)
Gender	−0.07*	−0.07*	−0.07*	−0.07**	−0.08**
	(0.03)	(0.03)	(0.03)	(0.03)	(0.03)
Elite	0.20***	0.22***	0.19***	0.24***	0.26***
	(0.06)	(0.05)	(0.05)	(0.04)	(0.04)
N(articles)	0.48***	0.44***	0.46***	0.44***	0.42***
	(0.04)	(0.04)	(0.04)	(0.04)	(0.04)
Variety	0.18***	0.28***	0.32***	0.36***	0.41***
	(0.04)	(0.03)	(0.03)	(0.03)	(0.03)
Balance	−0.15***	−0.22***	−0.26***	−0.30***	−0.33***
	(0.02)	(0.02)	(0.02)	(0.02)	(0.02)
Disparity	−0.14***	−0.12***	−0.11***	−0.12***	−0.11***
	(0.02)	(0.02)	(0.02)	(0.02)	(0.02)
Novelty	−0.02	0.02	0.04*	0.03*	0.03*
	(0.02)	(0.02)	(0.02)	(0.02)	(0.02)
2009	0.03	−0.03	−0.06	−0.05	−0.01
	(0.05)	(0.05)	(0.04)	(0.04)	(0.04)
2010	0.03	−0.03	−0.06	−0.07	−0.06
	(0.05)	(0.05)	(0.04)	(0.04)	(0.04)
2011	−0.04	−0.05	−0.07	−0.07	−0.03
	(0.05)	(0.05)	(0.04)	(0.04)	(0.04)
2012	0.03	−0.02	−0.05	−0.07	−0.03
	(0.05)	(0.05)	(0.04)	(0.04)	(0.04)
Adj. R^2	0.46	0.51	0.54	0.56	0.58
Num. Obs.	2251	3074	3591	3894	4097

$^{***}p < 0.001$; $^{**}p < 0.01$; $^{*}p < 0.05$

Linear regression models for the psychology sample with different early career lengths. Additional cases are suppressed.
