# Supplementary material for: Benefits and detriments of interdisciplinarity on early career scientists’ performance. An author-level approach for U.S. physicists and psychologists
Source: PLoS One. 2022 Jun 30;17(6):e0269991. doi: 10.1371/journal.pone.0269991 (PMC9246137; doi:10.1371/journal.pone.0269991)
Supplement: S3 File — (PDF) [file pone.0269991.s003.pdf]

S3 Robustness Check II: Varying citation window

As a third robustness check, we changed the duration of our citation window from three to five years (S3.1). To account for the longer duration of the citation window, we exclude ECRs who received their PhD after 2010.

Compared to our main models, most of our explanatory variables are robust for the longer citation window and the effects are similar in strength and significance.

Table S3.1. Main models with a five year citation window.

	physics sample	psychology sample
(Intercept)	5.01***	4.27***
	(0.04)	(0.04)
Gender	0.05	−0.04
	(0.05)	(0.03)
Elite	0.41***	0.29***
	(0.05)	(0.05)
N(articles)	0.81***	0.47***
	(0.03)	(0.05)
Variety	0.10**	0.39***
	(0.03)	(0.05)
Balance	−0.34***	−0.32***
	(0.02)	(0.02)
Disparity	−0.05	−0.12***
	(0.03)	(0.03)
Novelty	0.10***	0.04
	(0.02)	(0.02)
2009	−0.03	0.01
	(0.05)	(0.04)
2010	−0.05	−0.06
	(0.05)	(0.04)
Adj. R^2	0.56	0.57
AIC	6624.90	6040.77
Log. Lik.	−3301.45	−3009.38
Num. Obs.	2387	2440

$^{***}p < 0.001$; $^{**}p < 0.01$; $^{*}p < 0.05$

Results of the main models for physics and psychology with a five year citation window and possible career starts from 2008-2010.
