# Supplementary material for: Benefits and detriments of interdisciplinarity on early career scientists’ performance. An author-level approach for U.S. physicists and psychologists
Source: PLoS One. 2022 Jun 30;17(6):e0269991. doi: 10.1371/journal.pone.0269991 (PMC9246137; doi:10.1371/journal.pone.0269991)
Supplement: S4 File — (PDF) [file pone.0269991.s004.pdf]

S4 Robustness Check III: Variation in novelty range

To show that our model is robust for a longer novelty range, which we shortened in our main model to contain a larger sample, we provide the same model but with less cases and a five year *novelty* range in Table S4.1. As a consequence, the number of observation shrinks. Interestingly, the effect size of novelty increases in case of physics, indicating its robustness. At the same time, the loss of significance for psychology shows that our measure is not robust when controlled for a longer timeframe.

Table S4.1. Novelty range robustness.

	physics sample	psychology sample
(Intercept)	4.36^{***}	3.47^{***}
	(0.04)	(0.04)
Gender	0.05	-0.09^{**}
	(0.05)	(0.03)
Elite	0.42^{***}	0.24^{***}
	(0.05)	(0.05)
N(articles)	0.79^{***}	0.45^{***}
	(0.04)	(0.05)
Variety	0.10^{***}	0.36^{***}
	(0.03)	(0.04)
Balance	-0.33^{***}	-0.35^{***}
	(0.03)	(0.03)
Disparity	0.07^{*}	-0.08^{**}
	(0.03)	(0.03)
Novelty	0.11^{***}	0.03
	(0.02)	(0.02)
2011	0.02	0.03
	(0.05)	(0.04)
2012	0.03	0.04
	(0.05)	(0.04)
Adj. R^2	0.57	0.58
AIC	6557.82	6174.02
Log. Lik.	−3267.91	−3076.01
Num. Obs.	2372	2490

$^{***}p < 0.001$; $^{**}p < 0.01$; $^{*}p < 0.05$

Linear regression models for the psychology sample with a novelty range of five years instead of three.
