# Supplementary material for: Benefits and detriments of interdisciplinarity on early career scientists’ performance. An author-level approach for U.S. physicists and psychologists
Source: PLoS One. 2022 Jun 30;17(6):e0269991. doi: 10.1371/journal.pone.0269991 (PMC9246137; doi:10.1371/journal.pone.0269991)
Supplement: S5 File — (PDF) [file pone.0269991.s005.pdf]

S5 Illustrating key explanatory variables

This sections illustrates how we constructed our key explanatory variables. For that purpose, we use a small, fictitious sample of authors and articles. We start with $Author_1$ who published four articles $article_{1,1}$ - $article_{1,4}$ with the following classifications:

- $article_{1,1}$: a, b
- $article_{1,2}$: a, b, c
- $article_{1,3}$: c, d
- $article_{1,4}$: b, d

$Author_1$'s *variety* is four, i.e., the number of different classifications (a, b, c, d) across all articles.

Next, we look at the distribution of classifications and how often each classification occurs within $Author_1$'s articles. Classification a, c and d each occur twice while b occurs three times. The proportions p_i for each classification are calculated by dividing the number of occurrences for each classification by the total number of occurring classifications, so 2/9 for a, c and d, and 3/9 for b. Together with the *variety* of 4 from above, we can calculate the *balance*, which in this case (see equation S5.1) is 0.987.

$$\begin{aligned} balance &= -\frac{1}{ln(n_r)} \sum_i p_i \cdot ln(p_i) \\ &= -\frac{1}{ln(4)} \cdot (2/9 \cdot ln(2/9) + ... + 2/9 \cdot ln(2/9)) \\ &= 0.987 \end{aligned} \tag{S5.1}$$

For *disparity*, we need additional authors to which we compare $Author_1$. We therefore constructed a small artificial sample consisting of authors $Author_2$ - $Author_6$ and a small set of associated articles $article_{2,n}$ - $article_{6,n}$. Table S5.1 shows the full constructed sample, including $Author_1$.

From this sample, we then construct a $n \times m$ matrix S5.2. Therein, each row represents an author that contains a 1 for each classification if they published an article with the respective classification and a 0 otherwise.

From this $n \times m$ matrix, we can finally construct the co-occurrence matrix shown in table S5.3 that indicates how often a given combination of classifications occurs together.

Given the co-occurrence matrix, we can calculate the cosine distance for every combination of classifications within our dataset. For our example case, $Author_1$, we need the cosine distances (see equation S5.2) for the combinations $(a, b), (a, c), (a, d), (b, c), (b, d)$, and (c, d), which are $0.694, 0.898, 0.907, 0.667, 0.925$ and 0.841 respectively.

$$d_{ij} = 1 - s_{ij} \tag{S5.2}$$

If we insert these values into equation S5.3 we end up with a *disparity* value of 0.411 for $Author_1$.

$$disparity = \frac{1}{n_r(n_r - 1)} \sum_{ij} d_{ij} \tag{S5.3}$$

For our final main explanatory variable, *novelty*, we again look at the above-constructed sample for all fictitious authors; keep in mind, however, that

Table S5.1. Constructed author and article sample.

Author	Article	Classification						
		a	b	c	d	e	f	g
$Author_1$	$article_{1,1}$	x	x					
	$article_{1,2}$	x	x	x				
	$article_{1,3}$			x	x			
	$article_{1,4}$		x		x			
$Author_2$	$article_{2,1}$		x		x			
	$article_{2,2}$				x			x
	$article_{2,3}$		x					x
$Author_3$	$article_{3,1}$	x		x				
	$article_{3,2}$	x			x			
	$article_{3,3}$			x		x		
	$article_{3,4}$			x			x	
	$article_{3,5}$	x		x				
$Author_4$	$article_{4,1}$		x		x			
	$article_{4,2}$		x		x			x
$Author_5$	$article_{5,1}$			x			x	x
$Author_6$	$article_{6,1}$	x			x			
	$article_{6,2}$	x			x			

Illustrative example with fictitious authors, and associated articles and classifications.

Table S5.2. $n \times m$ **matrix of authors and classifications.**

Author	Classification						
	a	b	c	d	e	f	g
$Author_1$	1	1	1	1	0	0	0
$Author_2$	0	1	0	1	0	0	1
$Author_3$	1	0	1	1	1	1	0
$Author_4$	0	1	0	1	0	0	1
$Author_5$	0	0	1	0	0	1	1
$Author_6$	1	0	0	1	0	0	0

$n \times m$ matrix of authors and classifications, where each row represents an author and each column represents a classification.

Table S5.3. $m \times m$ **co-occurrence matrix.**

	a	b	c	d	e	f	g
a	3	1	2	3	1	1	0
b	1	3	1	3	0	0	2
c	2	1	3	2	1	2	1
d	3	3	2	5	1	1	2
e	1	0	1	1	1	1	0
f	1	0	2	1	1	2	1
g	0	2	1	2	0	1	3

$m \times m$ matrix that shows how often two classifications occur together.

disparity compares a given author to articles published **during** their early career. In contrast, *novelty* compares them to articles published **before** their early career. For a given article, we first need to calculate the number of expected articles for all combinations this given article is associated with (see equation S5.4).

$$N_{expected_{ij}} = \frac{c_i c_j}{N} \tag{S5.4}$$

$Article_{1,2}$, for example, has more than one classification combination, i.e., $(a, b), (a, c)$, and (b, c). For the combination (a, b), the expected number is calculated by multiplying the number of articles containing classification a with the number of articles containing classification b and dividing the product by the total number of articles (17). With 7 articles having classification a and 7 articles having classification b, the expected number of articles with the combination (a, b) would be $\frac{7\times7}{18} = 2.882$. To derive *novelty*, we also need the number of articles that we actually observe with the given combination, which is 2 before we can insert the values into equation S5.5.

$$novelty = 1 - \frac{N_{observed}}{N_{expected}} \tag{S5.5}$$

The *novelty* value therefore is $1 - \frac{2}{2.882} = 0.306$ The *novelty* values of the other combinations present within $article 1, 2$, (a, c) and (b, c) are -0.041 and 0.653 respectively. Please note that every article is assigned the highest *novelty* value of its combinations. The *novelty* values for all articles, as well as the mean percentiles per author in the constructed samples, are presented in table S5.4.

Table S5.4. Article level novelty and percentile values.

author	article	novelty	percentile (article)	mean percentile (author)
$Author_1$	$article_{1,1}$	0.306	0.824	0.765
	$article_{1,2}$	0.653	0.941	
	$article_{1,3}$	0.730	1.000	
	$article_{1,4}$	-0.079	0.294	
$Author_2$	$article_{2,1}$	-0.079	0.294	0.343
	$article_{2,2}$	0.056	0.559	
	$article_{2,3}$	-0.214	0.176	
$Author_3$	$article_{3,1}$	-0.041	0.441	0.353
	$article_{3,2}$	0.190	0.706	
	$article_{3,3}$	-1.429	0.088	
	$article_{3,4}$	-1.429	0.088	
	$article_{3,5}$	-0.041	0.441	
$Author_4$	$article_{4,1}$	-0.079	0.294	0.426
	$article_{4,2}$	0.056	0.559	
$Author_5$	$article_{5,1}$	0.393	0.882	0.882
$Author_6$	$article_{6,1}$	0.190	0.706	0.706
	$article_{6,2}$	0.190	0.706	

Article-level novelty and percentile values for the constructed sample.
