# Supplementary material for: Benefits and detriments of interdisciplinarity on early career scientists’ performance. An author-level approach for U.S. physicists and psychologists
Source: PLoS One. 2022 Jun 30;17(6):e0269991. doi: 10.1371/journal.pone.0269991 (PMC9246137; doi:10.1371/journal.pone.0269991)
Supplement: S6 File — (PDF) [file pone.0269991.s006.pdf]

S6 List of elite universities

The top 10 universities for physics and psychology in 2020 were retrieved from the U.S. News & World Report Rankings 2020. We selected the ten highest-ranked U.S. universities from the best universities for physics and psychology worldwide. The institutions we denoted as elite departments are presented in Table S6.

Table S6. Top 10 universities.

physics	Rank	psychology	Rank
Massachusetts Institute of Technology	1	Harvard University	1
Stanford University	2	Stanford University	2
University of California Berkeley	3	Columbia University	3 (tie)
Harvard University	4	Yale University	3 (tie)
The University of Chicago	5	University of California Los Angeles	4
California Institute of Technology	6	University of Pennsylvania	5
Princeton University	7	University of Michigan, Ann Arbor	6
Columbia University	8	Duke University	7
University of Maryland College Park	9	The University of North Carolina at Chapel Hill	8
University of Washington	10 (tie)	University of Pittsburgh	9
Yale University	10 (tie)	New York University	10

Top 10 U.S. universities by field.
