# Supplementary material for: Benefits and detriments of interdisciplinarity on early career scientists’ performance. An author-level approach for U.S. physicists and psychologists
Source: PLoS One. 2022 Jun 30;17(6):e0269991. doi: 10.1371/journal.pone.0269991 (PMC9246137; doi:10.1371/journal.pone.0269991)
Supplement: S7 File — (PDF) [file pone.0269991.s007.pdf]

S7 Regression diagnostics

Table S7.1. GVIF for both samples.

	physics	psychology
Gender		
Elite		
N(Articles)	3.37	5.46
Variety	2.80	4.70
Balance	1.74	1.58
Disparity	1.30	1.28
Novelty	1.09	1.15
Career Start		

Generalized variance inflation factors (GVIF) for the physics and psychology sample.

Table S7.2. BP tests for both samples.

	physics	psychology
statistic	134.20	98.99
p-value	0.00	0.00
df	11.00	11.00

Breusch-Pagan (BP) tests for the physics and psychology sample.
