# Supplementary material for: Benefits and detriments of interdisciplinarity on early career scientists’ performance. An author-level approach for U.S. physicists and psychologists
Source: PLoS One. 2022 Jun 30;17(6):e0269991. doi: 10.1371/journal.pone.0269991 (PMC9246137; doi:10.1371/journal.pone.0269991)
Supplement: S8 File — (PDF) [file pone.0269991.s008.pdf]

S8 Robustness Check IV: Negative binomial regression model

We estimated negative binomial regression models with total citations as the dependent variable as a third robustness check. We chose this approach over the poisson model to account for overdispersion present in citations. The dispersion index measured as $D = \frac{S^2}{X}$ is 621.08 for the physics sample and 327.30 for the psychology sample, indicating a high degree of overdispersion for both disciplines. As we included only researchers with at least one publication, there was no need to account for excess zeros.

Looking at the results in table S8, we see that most effects remain stable. There are, however, four notable exceptions. Most notably, *variety* changes signs from positive to negative in our physics sample, but remains highly significant. Furthermore, disparity gets weakly significant. In case of psychology, the gender effect loses its strength and is now only weakly significant. At the same time, novelty looses its significance. These findings stem from a different conception of the dependent variable, but might also hint that there might be more complex, underlying mechanisms linking IDR and novelty to research impact. However, as these models perform worse according to the *AIC* and *loglikelihood*, we decided to stick with the interpretation of the linear models.

Table S8. Results of the negative binomial regressions, with total citation count as dependent variable.

	physics sample	psychology sample
(Intercept)	4.79***	3.77***
	(0.03)	(0.03)
Gender	−0.01	−0.01
	(0.04)	(0.03)
Elite	0.42***	0.23***
	(0.03)	(0.04)
N(articles)	0.91***	0.63***
	(0.03)	(0.03)
Variety	−0.06**	0.26***
	(0.02)	(0.03)
Balance	−0.22***	−0.22***
	(0.02)	(0.02)
Disparity	0.06***	−0.09***
	(0.02)	(0.02)
Novelty	0.09***	0.00
	(0.02)	(0.01)
2009	0.03	0.04
	(0.04)	(0.04)
2010	0.03	0.02
	(0.05)	(0.04)
2011	0.01	0.17***
	(0.05)	(0.04)
2012	0.11*	−0.03
	(0.05)	(0.04)
AIC	47821.70	40416.65
Log. Lik.	−23897.85	−20195.33
Num. Obs.	4003	4097

$^{***}p < 0.001$; $^{**}p < 0.01$; $^{*}p < 0.05$

Results of the negative binomial regressions on ECRs research impact. All non-dummy explanatory variables have been standardized before modeling.
