# Supplementary material for: Benefits and detriments of interdisciplinarity on early career scientists’ performance. An author-level approach for U.S. physicists and psychologists
Source: PLoS One. 2022 Jun 30;17(6):e0269991. doi: 10.1371/journal.pone.0269991 (PMC9246137; doi:10.1371/journal.pone.0269991)
Supplement: S9 File — (PDF) [file pone.0269991.s009.pdf]

S9 Robustness check V: Interaction between the main effects

The interaction plots presented in Fig S9 show the interactions between the number of articles and *variety*, as well as the interaction between the number of articles and *balance* both for the physics and psychology sample. The effects of the mentioned explanatory interdisciplinarity variables on the number of citations received, depends heavily on the number of articles published, both in the physics and psychology sample.

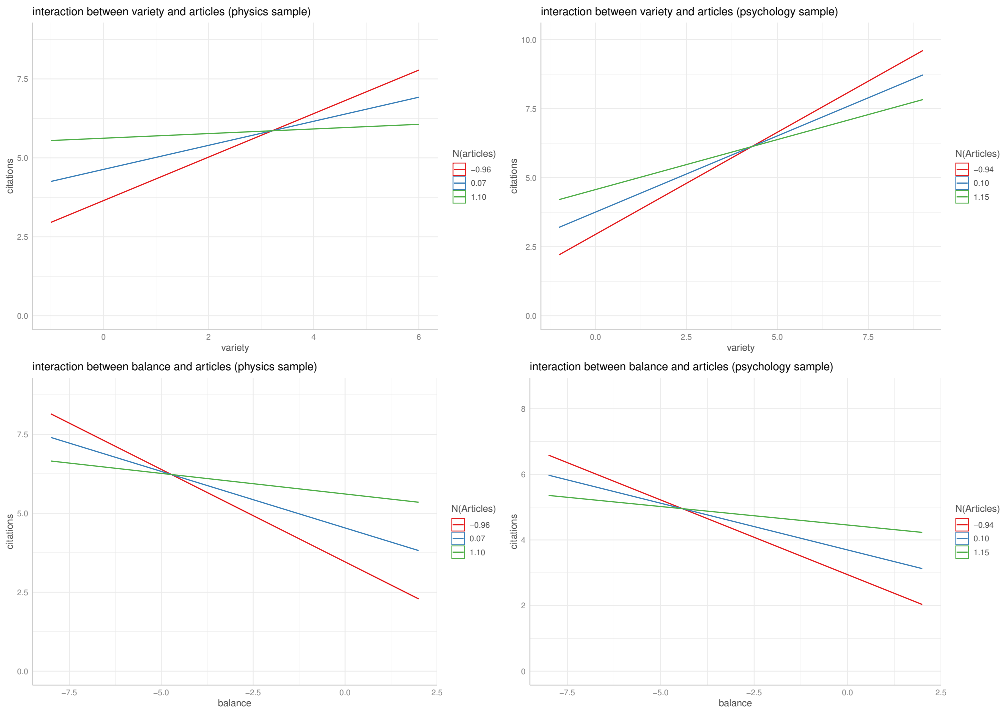

Fig S9. Interactions. Interactions between variety/balance and N(articles) for both samples.
