# Supplementary material for: Benefits and detriments of interdisciplinarity on early career scientists’ performance. An author-level approach for U.S. physicists and psychologists
Source: PLoS One. 2022 Jun 30;17(6):e0269991. doi: 10.1371/journal.pone.0269991 (PMC9246137; doi:10.1371/journal.pone.0269991)
Supplement: S10 File — (PDF) [file pone.0269991.s010.pdf]

S10 Descriptives

Table S10. Descriptives.

	physics							psychology						
	mean	std	min	25%	50%	75%	max	mean	std	min	25%	50%	75%	max
Variety	10.06	8.25	2.00	5.00	7.00	12.00	58.00	8.70	7.11	2.00	4.00	7.00	10.00	67.00
Balance	0.89	0.11	0.16	0.85	0.92	0.96	1.00	0.92	0.07	0.41	0.88	0.93	0.97	1.00
Disparity	0.16	0.05	0.04	0.13	0.15	0.19	0.54	0.21	0.06	0.07	0.17	0.19	0.24	0.74
Novelty	62.30	18.63	1.00	48.33	64.00	78.50	100.00	65.57	17.30	1.00	56.00	68.22	78.00	100.00
Elite	0.24	0.42	0.00	0.00	0.00	0.00	1.00	0.10	0.30	0.00	0.00	0.00	0.00	1.00
Gender	0.17	0.38	0.00	0.00	0.00	0.00	1.00	0.56	0.50	0.00	0.00	1.00	1.00	1.00
N(Articles)	16.11	15.17	2.00	5.00	11.00	21.00	91.00	10.27	10.66	2.00	4.00	7.00	12.00	91.00
N(Citations)	12.65	14.56	0.00	5.00	8.47	15.07	274.00	6.84	10.30	0.00	3.20	5.29	8.00	347.10

Descriptive statistics for both samples.
